# Age and decisions to limit life support for patients with acute lung injury: a prospective cohort study

**DOI:** 10.1186/cc13890

**Published:** 2014-05-26

**Authors:** Alison E Turnbull, Bryan M Lau, A Parker Ruhl, Pedro A Mendez-Tellez, Carl B Shanholtz, Dale M Needham

**Affiliations:** 1 The Outcomes After Critical Illness and Surgery (OACIS) Group, Johns Hopkins University School of Medicine, 1830 E. Monument St. 5th floor, Baltimore, MD 21205, USA; 2 The Division of Pulmonary and Critical Care Medicine, Johns Hopkins University School of Medicine, 1830 E. Monument St. 5th floor, Baltimore, MD 21205, USA; 3 Department of Epidemiology, Johns Hopkins University Bloomberg School of Public Health, 615 N. Wolfe Street, Baltimore, MD 21205, USA; 4 Critical Care Medicine Department, National Institutes of Health, 10 Center Drive, Room 2C145, Bethesda, MD 20892-1662, USA; 5 Department of Anesthesiology and Critical Care Medicine, Johns Hopkins University School of Medicine, 600 N. Wolfe St., Baltimore, MD 21287, USA; 6 The Division of Pulmonary and Critical Care Medicine, University of Maryland, 110 South Paca Street, Baltimore, MD 21201, USA; 7 Department of Physical Medicine and Rehabilitation, Johns Hopkins University School of Medicine, 600 N. Wolfe St., Baltimore, MD 21287, USA

## Abstract

**Introduction:**

The proportion of elderly Americans admitted to the intensive care unit (ICU) in the last month of life is rising. Hence, challenging decisions regarding the appropriate use of life support are increasingly common. The objective of this study was to estimate the association between patient age and the rate of new limitations in the use of life support, independent of daily organ dysfunction status, following acute lung injury (ALI) onset.

**Methods:**

This was a prospective cohort study of 490 consecutive patients without any limitations in life support at the onset of ALI. Patients were recruited from 11 ICUs at three teaching hospitals in Baltimore, Maryland, USA, and monitored for the incidence of six pre-defined limitations in life support, with adjustment for baseline comorbidity and functional status, duration of hospitalization before ALI onset, ICU severity of illness, and daily ICU organ dysfunction score.

**Results:**

The median patient age was 52 (range: 18 to 96), with 192 (39%) having a new limitation in life support in the ICU. Of patients with a new limitation, 113 (59%) had life support withdrawn and died, 53 (28%) died without resuscitation, and 26 (14%) survived to ICU discharge. Each ten-year increase in patient age was independently associated with a 24% increase in the rate of limitations in life support (Relative Hazard 1.24; 95% CI 1.11 to 1.40) after adjusting for daily ICU organ dysfunction score and all other covariates.

**Conclusions:**

Older critically ill patients are more likely to have new limitations in life support independent of their baseline status, ICU-related severity of illness, and daily organ dysfunction status. Future studies are required to determine whether this association is a result of differences in patient preferences by age, or differences in the treatment options discussed with the families of older versus younger patients.

## Introduction

The proportion of older Americans utilizing intensive care in the last month of life has steadily increased over the past two decades [1,2]. Moreover, the proportion of the US population over 65 years old is expected to double between 2000 and 2030 [3], with increasing demand for intensive care [2,4,5]. Despite calls for increased discussion and documentation of end-of-life wishes, such issues are frequently raised only after patients are hospitalized with acute conditions requiring ICU admission.

The Study to Understand Prognoses and Preferences for Outcomes and Risks of Treatments (SUPPORT), which completed enrollment two decades ago, examined the relationship between patient age and decisions to limit the use of life support for patients hospitalized with nine life-threatening diagnoses including acute respiratory failure [6]. SUPPORT investigators found that, after adjusting for patients’ baseline function, preferences, and prognosis, older age was associated with higher rates of withholding mechanical ventilation, surgery, and dialysis [7]. However, the SUPPORT study could not evaluate whether older patients had higher rates of withholding because they experienced greater organ dysfunction than younger patients.

In 2003, Cook and colleagues examined predictors of withdrawing ventilator support using longitudinal data and found no association with age after controlling for physician predictions about both short-term and long-term patient outcomes [8]. To further investigate this question, we analyzed a prospective cohort of critically ill patients with acute lung injury (ALI), which includes longitudinal data on daily organ dysfunction status throughout their ICU stay. We hypothesized that although organ dysfunction during hospitalization is likely to impact decisions to limit life support, patient age is directly associated with the rate of new limitations independent of organ dysfunction.

## Materials and methods

### Study cohort

Between October 2004 and October 2007, mechanically ventilated patients who met the American–European Consensus criteria for ALI [9] were consecutively enrolled from 13 ICUs at four teaching hospitals in Baltimore, MD, USA [10]. The parent study’s primary aim was to evaluate the longer-term outcomes of ALI survivors with a particular focus on the effect of low tidal volume ventilation and other critical care therapies [11]. All patients in participating ICUs were evaluated daily for the presence of ALI by trained research personnel along with study investigators. Only patients who were able to understand or speak English were considered for consent.

Patients in neurologic specialty ICUs were excluded to avoid enrolling patients with head trauma or primary neurologic disease. Other key exclusion criteria were: pre-existing illness with a life expectancy of <6 months (based on the judgment of the patient’s medical team); pre-existing cognitive impairment (based on documentation in the medical record); no fixed address; transfer to a study site ICU with pre-existing ALI of >24 hours duration; >5 days of mechanical ventilation before ALI; and a pre-existing limitation in life support at the time of study eligibility (except for a sole order for no cardiopulmonary resuscitation (CPR) in the event of a cardiac arrest). For this analysis we excluded research participants who had a sole no-CPR order at ALI onset (*n* = 24), so that all participants had no limitations in life support at ALI onset. In addition, participants recruited from two ICUs at the Veterans Affairs hospital study site (*n* = 6) were excluded because medical records were inaccessible for independent verification of limitations of life support at the time of this analysis.

Consequently, a total of 490 patients from 11 ICUs were available for this analysis of patients’ index ICU admission for ALI. The institutional review boards of Johns Hopkins University and all participating study sites approved this research, with a waiver of consent allowing observational data to be collected from the medical records during patients’ hospitalization (see Acknowledgements).

### Primary outcome

Patient medical records were evaluated by trained study personnel using a standardized data collection form. A limitation in life support was defined as a physician order or a clinical note documenting any of the following: no CPR; do not reintubate; no vasopressors; no hemodialysis; do not escalate care; or other limitation (for example, comfort care only). These limitations were considered in this analysis when initiated either with or without a concomitant decision to withdraw life support.

### Primary exposure

The primary exposure for this analysis was patient age at the time of study enrollment. To investigate for a potential nonlinear relationship between age and the rate of limitations in life support, we evaluated both linear spline terms and age functions using fractional polynomials in multivariable models, and found no evidence of nonlinearity. Hence, age was modeled as a continuous variable, with results of the analysis reported for each 10-year increase within the age distribution of the cohort.

### Covariates

The following baseline patient characteristics were hypothesized to be potentially associated with the use of life support: sex, race (white vs. nonwhite) [12,13], history of cancer (all previous diagnoses of leukemia, lymphoma, solid tumors with metastatic disease, as well as solid tumors without metastatic disease in the last 5 years) [14], Charlson Comorbidity Index [15], Functional Comorbidity Index [16] as a measure of functional status before hospitalization, days of hospitalization before ALI onset, and Acute Physiology and Chronic Health Evaluation II severity of illness score [17] at ICU admission. Two organizational factors, hospital study site (site 1, 2, or 3) and ICU type (medical vs. surgical), were also considered. Finally, we also included the severity of daily dysfunction, as a continuous variable, in each of six organ systems (respiratory, circulatory, renal, hematologic, hepatic, and central nervous system) during the ICU stay using the Sequential Organ Failure Assessment (SOFA) score, which gives a higher score (range: 0 to 4) for greater dysfunction [18]. SOFA scores were missing for nine of 8,673 (0.10%) days of observation. These scores were imputed by multiple imputation and included in analysis.

### Statistical analysis

All patients were evaluated in this analysis until one of three mutually exclusive events occurred: cardiac arrest, discharge from the ICU, or a limitation in the use of life support (as previously defined). No patients were lost to follow-up, with all experiencing one of these three events. A cause-specific hazard approach was used to separately estimate the impact of age on limitations in life support and the other two competing events as well as the cumulative incidence of each event over 28 days in the ICU setting [19-21].

To estimate the overall association between patient age and the rate of limitations in life support, we fit an initial flexible parametric survival model [22-24] using restricted cubic splines of the Weibull distribution with four degrees of freedom, adjusting for all baseline covariates. In this initial model, each organ system component of the SOFA score from the day of ALI onset was included as a baseline covariate. Next, to estimate the direct association between patient age and the rate of limitations in life support, independent of organ dysfunction during the ICU stay, we added each of the six components of the daily SOFA score as a time-varying covariate to the initial model. Relative hazards (RHs) and 95% confidence intervals (CIs) are reported.

Finally, for illustrative purposes, we derived and plotted the 28-day cumulative incidence function from the initial model to estimate the probability of each event (cardiac arrest, limitation in life support, and ICU discharge) for a prototypical patient with identical baseline characteristics (median values for all baseline continuous covariates and mode values for all binary covariates), but with varying ages [19,25]. Analyses were conducted using STATA 11.0 (StataCorp, College Station, TX, USA).

## Results

**Figure 1:**
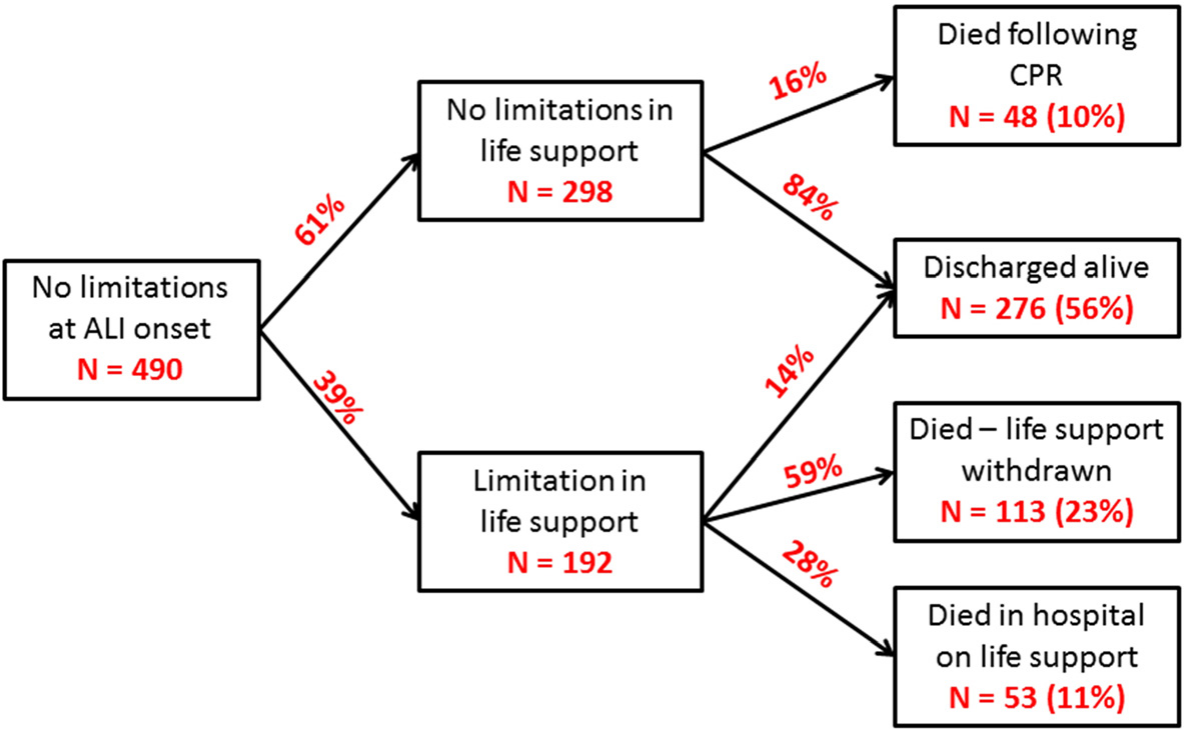
**Patient trajectories and outcomes following acute lung injury onset.** Percentages in boxes represent the proportion of all 490 patients with the characteristic or outcome. Percentages along the arrows refer to the proportion of patients who transitioned to the outcome described in the box where the arrow terminates. Proportions do not sum to 100% due to rounding. ALI, acute lung injury; CPR, cardiopulmonary resuscitation.

Patients included in this analysis ranged in age from 18 to 96 years old, with a median age of 52 (interquartile range 42 to 62), while patients excluded because of a pre-existing no-CPR order at the time of ALI onset had a median age of 62 (interquartile range 49 to 79). Of all nonwhite patients (41%), 95% were African-American (Table 1). One-half of all patients had been hospitalized for <3 days before ALI onset. During follow-up, 192 patients (39%) had a limitation in life support before ICU discharge or cardiac arrest, occurring a median 7.5 days (interquartile range 3 to 16 days) after ALI onset. Of these patients, 113 (59%) had life support withdrawn and died, 53 (28%) died without resuscitation efforts and 26 (14%) survived to ICU discharge (Figure 1). Patients aged 70 to 79 years old had the shortest median time from ALI onset to a limitation in life support, and patients ≥80 years old were most likely to have a limitation in life support before ICU discharge or cardiac arrest (Table 2).

**Table 1:** Patient demographics and outcomes, by first event following acute lung injury onset

		**By first event**		
	**All patients ( *N *** **= 490)**	**Discharge ( *n *** **= 235)**	**Cardiac arrest ( *n *** **= 63)**	**Limitation in life support ( *n *** **= 192)**
Age	52 (42 to 62)	48 (40 to 59)	50 (41 to 63)	56 (48 to 66)
Female	43.5	46.0	41.3	41.1
Nonwhite	40.8	39.2	46.0	41.2
Oncology comorbidity	18.4	15	17	22
Charlson Comorbidity Index	2 (1 to 4)	1 (0 to 3)	3 (1 to 5)	3 (1 to 4)
Functional Comorbidity Index	1 (1 to 3)	1 (1 to 3)	1 (0 to 3)	1 (1 to 3)
Study site hospital				
1	38.0	31.9	42.9	43.8
2	31.0	31.9	33.3	29.2
3	31.0	36.2	23.8	27.1
Days in hospital before ALI onset	2 (1 to 6)	2 (1 to 5)	2 (1 to 10)	3 (1 to 7)
Medical ICU	80.4	74.9	84.1	85.9
APACHE II score at ICU admission	26 (20 to 33)	23 (19 to 29)	33 (22 to 38)	29 (22 to 35)
SOFA score at ALI onset	9 (7 to 12)	8 (5 to 10)	12 (8 to 14)	11 (8 to 14)
Survival to ICU discharge	56.3	100.0	23.8	13.5

Data presented as median (interquartile range) or percentage. ALI, Acute lung injury; APACHE, Acute Physiology and Chronic Health Evaluation; SOFA, Sequential Organ Failure Assessment.

**Table 2:** Selected patient characteristics and outcomes, by decade of age

		**Patient age**					
		**<40**	**40 to 49**	**50 to 59**	**60 to 69**	**70 to 79**	**≥80**
Number of patients (n)		94	125	236	74	45	26
Charlson Comorbidity Index		1 (0 to 4)	2 (1 to 4)	2 (1 to 4)	3 (1 to 4)	3 (2 to 4)	3 (2 to 4)
Functional Comorbidity Index		1 (0 to 1)	1 (0 to 2)	1 (0 to 3)	2 (1 to 3)	3 (2 to 4)	3 (1 to 4)
APACHE II score at ICU admission^a^		24 (20 to 33)	24 (19 to 33)	28 (21 to 33)	27 (20 to 34)	28 (20 to 36)	29 (21 to 35)
First event^b^							
Cardiac arrest		15	13	13	8	13	15
ICU discharge		60	58	41	43	38	23
Limitation in life support		26	30	45	49	49	62
Days from ALI onset to limitation in life support^c^		8 (6 to 19)	7 (3 to 19)	9 (3 to 16)	7 (3 to 13)	4 (2 to 10)	11 (4 to 16)

Data presented as median (interquartile range) or percentage, unless otherwise stated. ALI, acute lung injury; APACHE, Acute Physiology and Chronic Health Evaluation. ^a^APACHE II scores include points awarded for patient age. After removing these points, the median and interquartile range of scores for each age category are as follows: <40, 24 (20 to 33); 40 to 49, 23 (18 to 32); 50 to 59, 25 (18 to 31); 60 to 69, 22 (16 to 29); 70 to 79, 23 (15 to 30); ≥80, 23 (15 to 29). ^b^Proportions may not sum to 100% due to rounding. ^c^Among patients with a limitation in life support before ICU discharge or cardiac arrest.

**Figure 2:**
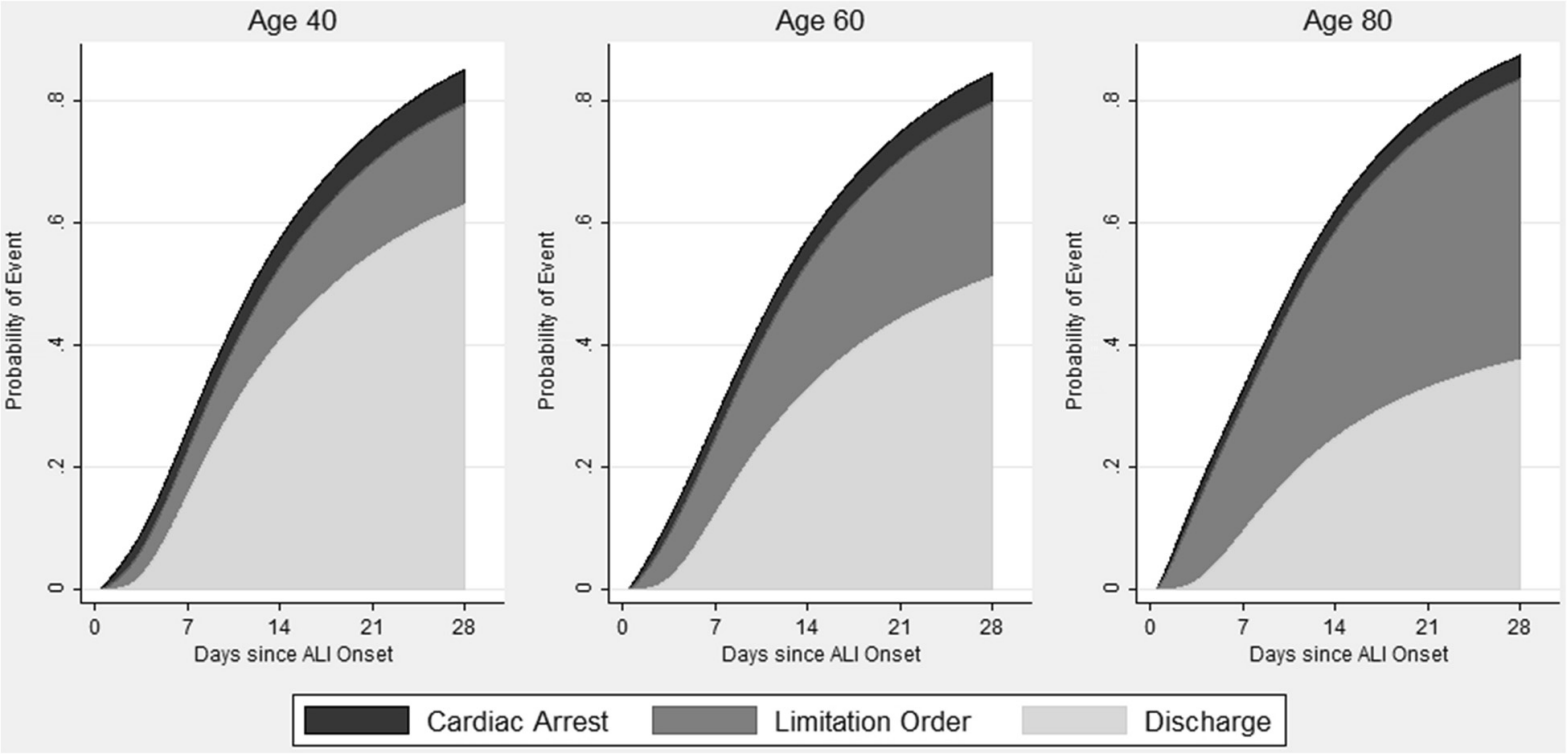
**Estimated probability of cardiac arrest, limitation in life support, and ICU discharge, by patient age.** Estimated probability of cardiac arrest, limitation in life support, or ICU discharge for a typical patient in this acute lung injury (ALI) cohort (male patient hospitalized for 2 days before ALI onset, without any oncology comorbidity in a medical ICU at hospital site 1 with median values for Acute Physiology and Chronic Health Evaluation II, Functional Comorbidity Index, Charlson Comorbidity Index, and Sequential Organ Failure Assessment scores) at the time of enrollment. The cumulative incidence of each outcome was obtained through estimation using the initial model containing all baseline covariates. The figure has been truncated at 28 days; hence, the cumulative probability of all events does not reach 100% because 11% of patients had their first event more than 28 days after ALI onset. From left to right, the panels present the estimated probability of events when the patient is age 40, 60, and 80.

In the initial survival model containing only baseline covariates, age was significantly associated with the rate of limitations in life support (RH, 1.32; 95% CI, 1.18 to 1.48), as was the hepatic component of the SOFA score (RH, 1.25; 95% CI, 1.08 to 1.44) (Table 3). For a prototypical patient, the cumulative probability of ICU discharge, cardiac arrest, and a limitation in life support during 28 days after ALI onset is displayed in Figure 2, with the estimated probability (95% CI) of a limitation in life support of 17% (9 to 24%) for a 40 year old, 29% (18 to 40%) for a 60 year old, and 46% (29 to 63%) for an 80 year old.

**Table 3:** **Cause-specific relative hazards**^**a**^**for limitation of life support in 490 patients with acute lung injury**

	**Baseline model**^**b**^		**Time-varying model**^**b**^	
**Patient demographic at enrollment**	**RH (95% CI)**	***P*** **value**	**RH (95% CI)**	***P*** **value**
Age (10-year increase)	1.32 (1.18 to 1.48)	0.001	1.24 (1.11 to 1.40)	0.001
Female	0.99 (0.73 to 1.34)	0.96	0.97 (0.71 to 1.32)	0.86
Nonwhite	1.23 (0.89 to 1.70)	0.21	1.37 (0.99 to 1.92)	0.06
Cancer history	1.18 (0.78 to 1.79)	0.42	1.59 (1.04 to 2.43)	0.03
Charlson Comorbidity Index	1.02 (0.96 to 1.09)	0.53	0.98 (0.92 to 1.05)	0.58
Functional Comorbidity Index	1.00 (0.90 to 1.10)	0.94	1.03 (0.93 to 1.14)	0.53
Study site hospital				
1 (reference group)	Reference	Reference	Reference	Reference
2	1.19 (0.81 to 1.77)	0.38	1.32 (0.90 to 1.94)	0.15
3	1.14 (0.75 to 1.73)	0.55	1.13 (0.76 to 1.69)	0.54
Days in hospital before ALI onset (1-day increase)	1.01 (0.99 to 1.02)	0.29	0.99 (0.98 to 1.00)	0.01
Medical ICU	1.92 (1.20 to 3.07)	0.007	1.87 (1.16 to 3.02)	0.01
APACHE II score at ICU admission	1.02 (0.99 to 1.04)	0.15	0.99 (0.97 to 1.01)	0.28
Components of SOFA score				
Respiratory	0.93 (0.75 to 1.15)	0.50	1.19 (1.02 to 1.40)	0.03
Cardiovascular	1.06 (0.96 to 1.17)	0.28	1.47 (1.33 to 1.63)	0.001
Hepatic	1.25 (1.08 to 1.44)	0.002	1.17 (1.03 to 1.33)	0.01
Coagulation	1.10 (0.96 to 1.27)	0.18	1.23 (1.07 to 1.41)	0.004
Renal	1.05 (0.93 to 1.18)	0.43	1.15 (1.03 to 1.29)	0.02
Neurologic	1.01 (0.90 to 1.14)	0.82	1.10 (0.98 to 1.22)	0.10

ALI, acute lung injury; APACHE, Acute Physiology and Chronic Health Evaluation; CI, confidence interval; RH, relative hazard; SOFA, Sequential Organ Failure Assessment. ^a^The relative hazard describes the relative risk of a new limitation in life support on a specific day in the ICU among patients who have not had a cardiac arrest or been discharged from the ICU. For example, a patient with a relative hazard of 1.59 has a 59% increased risk of having a limitation in life support that day compared with the reference patient if neither patient has a cardiac arrest or is discharged from the ICU. ^b^The baseline model was adjusted for each component of SOFA score on the day of ALI onset. The time-varying model was adjusted for each component of SOFA score on all days in the ICU prior to the day of first event. Both models are adjusted for sex, race, oncology comorbidity, Charlson Comorbidity Index, Functional Comorbidity Index, study site hospital, days in hospital before ALI onset, ICU type, and APACHE II score at ICU admission.

Adding the SOFA score as a time-varying covariate to the initial model with only baseline covariates modestly attenuated the relationship between patient age and the rate of limitations in life support (RH, 1.24; 95% CI, 1.11 to 1.40), with all components of the SOFA score except neurologic status significantly associated with the rate of limitations (Table 3). Older age was not significantly associated with the rate of ICU discharge (RH, 1.04; CI, 0.94 to 1.15) or cardiac arrest (RH, 0.86; CI, 0.70 to 1.05).

## Discussion

In this multisite, prospective cohort study of 490 patients with acute lung injury, each 10-year increase in patient age was associated with a 24% increase (95% CI 11 to 40%) in the rate of new limitations in life support, after accounting for differences in baseline status, ICU severity of illness, daily organ dysfunction, and the competing risks of ICU discharge and cardiac arrest. Adding longitudinal data on daily organ dysfunction status to the initial model with baseline patient and ICU data attenuated the association between age and the rate of limitations in life support, suggesting that organ dysfunction may be a mediator in the age-limitation relationship but does not fully explain the association.

Our findings expand upon the findings of prior studies that have evaluated the relationship between age and the limitation of life support [26], and suggest that factors arising after hospital admission, rather than only a patient’s status before hospitalization, are important for understanding new limitations in the ICU setting. A 2011 systematic review reported that age was one of the most consistent patient characteristics influencing decisions about end-of-life care [27]. However, most studies in this systematic review used scenario-based surveys that are vulnerable to social desirability bias, or cohort studies that did not include data on patients’ condition following hospital admission. New limitations in life support, sometimes made after days or weeks in the ICU, were significantly associated with organ dysfunction for five of the six separate organ systems evaluated daily, but not with baseline characteristics such as comorbidity or physical functional status.

Our results highlight that a patient’s resuscitation status at ICU admission should not be viewed as static. None of the 490 patients evaluated in this study had any limitation in life support while receiving mechanical ventilation at ALI onset in the ICU, but 39% had at least one limitation before cardiac arrest or ICU discharge. Very few patients with ALI are able to communicate or participate in clinical decision-making. New limitations occurring after enrollment in this study are thus likely to reflect decisions made by a surrogate decision-maker who is typically a close family member. These surrogates and physicians are faced with determining what treatments and interventions patients wish to receive based on past discussions and knowledge of the patient’s values and preferences. Without data on such discussions, we cannot infer whether the higher rate of limitations among older patients in this study reflects patients’ wishes, or surrogates’ and physicians’ assumptions about the preferences of older patients. This is consistent with Cook and colleagues’ 2003 study, which found that physician prediction regarding a patient’s preference for the use of life support was strongly associated with a decision to withdraw mechanical ventilation [8].

Are assumptions that older patients are less likely to elect life-supporting interventions correct? Two decades ago, older patients in the SUPPORT study were less likely to elect CPR than similar younger patients [28], but families and healthcare providers also underestimated older patients’ desire for aggressive life-sustaining treatments [29]. Smaller, more recent studies suggest that older patients in the United States are more likely to be asked about resuscitation status at hospital admission independent of illness severity [30], but are also less likely to receive end-of-life care consistent with their stated preferences [31]. The increased rate of limitations observed possibly reflects differences in long-held, generational attitudes about end-of-life care. However, a recent study of more than 118,000 long-term nursing home residents found that 40% of residents who wished to receive CPR at enrollment changed their minds within 5 years, suggesting that patient preferences change over time [32]. Finally, it is possible that life-sustaining interventions are more likely to be perceived as inappropriate in older patients. Reports of disproportionately aggressive treatment for patients who are unlikely to benefit are not uncommon among clinicians in Europe, Israel, and the United States [33,34].

There are potential limitations to this study. First, only 71 patients in the study cohort were more than 70 years old. This both limits our power to estimate the effect of age for older patients [35,36] and may explain why we did not observe an exponential increase in the rate of limitations among the oldest patients, as previously observed in the SUPPORT cohort [7]. Nonetheless, the cohort comprised consecutively admitted patients at 11 ICUs in three hospitals and the distribution of patient ages was nearly identical to a large cohort of ALI patients at US academic hospitals [37]. Second, our broad definition of a limitation in life support means our results are not directly comparable with previous studies that focus exclusively on the relationship between age and cardiopulmonary resuscitation preference, ICU admission, or decisions to withdraw mechanical ventilation. However, we believe that the broader definition used in this study better reflects the range of decisions made in an ICU setting about the use of life-sustaining interventions. Third, the study cohort excluded patients with cognitive dysfunction. The role of age in decisions about the use of life support may be quite different for these patients, and this is a fertile area for future research as the number of people with dementia climbs. Finally, our study was limited to academic, tertiary-care hospitals in a single city. The high in-hospital mortality observed in this study is consistent with prior ALI studies conducted in university-affiliated hospitals in North America [27,28,37,38], but geographic variability in the use of medical interventions at the end of life may make our results less generalizable to other settings [39,40].

## Conclusions

Older critically ill patients with ALI are more likely to have new limitations in life support in the ICU independent of other predictors, including baseline demographics, comorbidity, and functional status, and ICU severity of illness and daily organ dysfunction status. Further study is needed to determine whether the greater rate of limitations in life support for older patients is a reflection of barriers to end-of-life care discussions with families of younger patients, or of family or physician beliefs, which could be accurate or inaccurate, about the preferences of older patients.

## Key messages

● Patients’ resuscitation status during critical illness is dynamic. In this cohort of 490 patients with no limitations in life support at ALI onset, 39% had new limitations in life support before cardiac arrest or ICU discharge.

● Older patient age was associated with an increased rate of limitations in life support after adjusting for all baseline covariates and daily ICU organ dysfunction status.

● Higher rates of limitations in life support among older patients are not primarily a result of older patients’ baseline comorbidity or functional status, nor a result of greater organ dysfunction following ALI onset.

● Future studies should investigate whether higher rates of limitations among older patients are a result of differences in patient preferences by age, or differences in the treatment options discussed with the families of older versus younger patients.

## Abbreviations

ALI: acute lung injury; CI: confidence interval; CPR: cardiopulmonary resuscitation; RH: relative hazard; SOFA: Sequential Organ Failure Assessment; SUPPORT: Study to Understand Prognoses and Preferences for Outcomes and Risks of Treatments.

## Competing interests

This research was supported by the National Institutes of Health (Acute Lung Injury Specialized Centers of Clinically Oriented Research grant number P050 HL 73994). AET is supported by the Johns Hopkins University Sommer Scholars Program and a postdoctoral training grant from the National Institute on Aging, T32AG000247. The authors declare that they have no competing interests.

## Authors’ contributions

AET was responsible for the design of the study, data analysis and interpretation, and drafted the manuscript. BML was responsible for the design of the study, interpretation of the analyses, and critically revised the article for intellectual content. APR was responsible for the design of the study, interpretation of the analyses, and critical revision of the article for intellectual content. PAM-T was responsible for the study conception and design, interpretation of the analyses, critical revision and final approval of the manuscript. CBS was responsible for the study conception and design, interpretation of the analyses, critical revision and final approval of the manuscript. DMN was responsible for the study conception and design, interpretation of the analyses, as well as critical revision and final approval of the manuscript. All authors read and approved the final manuscript.
